# Daily Time-Use Patterns and Quality of Life in Parents: Protocol for a Pilot Quasi-Experimental, Nonrandomized Controlled Trial Using Ecological Momentary Assessment

**DOI:** 10.2196/54728

**Published:** 2024-05-31

**Authors:** Laura Altweck, Silke Schmidt, Samuel Tomczyk

**Affiliations:** 1 Department Health and Prevention Institute of Psychology University of Greifswald Greifswald Germany

**Keywords:** time-use, well-being, parents, ecological momentary assessment, feasibility, health-related quality of life, ambulatory assessment, work-family conflict, gender roles, mixed-methods, sex differences, stress

## Abstract

**Background:**

The gender gap in time use and its impact on health and well-being are still prevalent. Women work longer hours than men when considering both paid and unpaid (eg, childcare and chores) work, and this gender disparity is particularly visible among parents. Less is known about factors that could potentially mediate or moderate this relationship (eg, work-family conflict and gender role beliefs). Ecological momentary assessment (EMA) allows for the documentation of changes in momentary internal states, such as time use, stress, or mood. It has shown particular validity to measure shorter-term activities (eg, unpaid work) and is thus useful to address gender differences.

**Objective:**

The feasibility of the daily EMA surveys in a parent sample will be examined. The associations between time use, well-being, and stress will be examined, along with potential moderating and mediating factors such as gender, gender role beliefs, and work-family conflict. Finally, the act of monitoring one’s own time use, well-being, and stress will be examined in relation to, for example, the quality of life.

**Methods:**

We conducted a quasi-experimental, nonrandomized controlled trial with 3 data collection methods, namely, online questionnaires, EMA surveys, and qualitative interviews. The intervention group (n=64) will participate in the online questionnaires and EMA surveys, and a subsample of the intervention group (n=6-17) will also be invited to participate in qualitative interviews. Over a period of 1 week, participants in the intervention group will answer daily EMA surveys (4 times per day). In contrast, the control group (n=17) will only participate in the online questionnaires at baseline and after 1 week. The following constructs were surveyed: sociodemographic background (eg, age, gender, and household composition; baseline questionnaire); mediators and moderators (eg, gender role beliefs and work-family conflict; baseline and follow-up questionnaires); well-being, quality of life, and trait mindfulness (baseline and follow-up questionnaires); momentary activity and well-being, as well as state mindfulness (EMA); and feasibility (baseline and follow-up questionnaires as well as interviews). We anticipate that participants will regard the daily EMA as feasible. Particular daily time-use patterns (eg, high paid and unpaid workload) are expected to be related to lower well-being, higher stress, and health-related quality of life. These associations are expected to be moderated and mediated by factors such as gender, gender role beliefs, work-family conflict, and social support. Participants in the intervention group are expected to show higher values of mindfulness, well-being, health-related quality of life, and lower stress.

**Results:**

Patient recruitment started in November 2023 and ended in mid April 2024. Data analysis commenced in mid April 2024.

**Conclusions:**

This study aims to provide valuable insights into the feasibility of using EMAs and the potential benefits of activity tracking in various aspects of daily life.

**Trial Registration:**

Open Science Framework 8qj3d; https://osf.io/8qj3d

**International Registered Report Identifier (IRRID):**

PRR1-10.2196/54728

## Introduction

### Overview

Despite aspirations for gender equality, the “gender gap” continues to be evident in many areas of daily life, including everyday time use and the associated consequences for health and well-being. During the COVID-19 pandemic, this divergence was particularly evident as many women (eg, due to school closures and working from home) felt increased pressures from 2 sides, namely work and family [1]. Overall, time use is an important determinant of health and quality of life. For example, long work hours [2,3], physical and inactive lifestyles [4,5], and informal caregiving for family members [6] are associated with poorer quality of life.

Women, like men, now spend most of their day doing paid work; however, women also do most of the housework, childcare, and unpaid care work [7]. According to Coltrane [8], women’s share of housework is double that of men’s. The largest gender differences are seen among those with children of preschool age [9]. As a result, when paid work and this so-called unpaid work are summed, women (mean 6:44-9:13 hours) work significantly longer hours than men (mean 6:36-8:07 hours) [10]. In terms of leisure time, fathers report more free time than mothers, which is made up of longer uninterrupted periods of rest and is also less often followed by childcare [11]. Gender differences in time use and the impact on health and well-being have been well established in previous research [6,7,10,12,13]. Less is known about how gender and other factors could potentially mediate or moderate the relationship between time use and health and well-being.

### Theoretical Frameworks

There are several theoretical models that explain how people use their time and how this affects their quality of life. One of these theories is the conservation of resources theory [14], which states that people use their time in a way that preserves or enhances their resources (eg, energy, money, or social support). Instead, the time availability theory [15] states that time budgets are dependent on the availability of time and demands in a particular role, while the relative resources theory [16] assumes that time budgets are set depending on the relative power someone has in a relationship or organization. In addition, the work-family conflict model [17] states that time budgets are divided between work and family based on conflicts and demands. The gender perspective—which is often used to complement these theories—is concerned with how gender roles influence time use. For example, the theories of “doing gender” [18] or “gender performativity” [19] highlight how gender roles are constructed through actions and behaviors.

### Survey Methods of Time Use

Generally, diaries are the most common method to measure time use [20,21]. In some cases, self-reports are combined with objective physiological measurements; for example, sleep and wake times are recorded by a motion sensor and well-being is recorded in a diary throughout the day [22]. Ambulatory assessment, such as ecological momentary assessment (EMA), is the measurement of, for example, the mood, symptoms, and activities of individuals in real time and in their natural environment. An advantage of EMA is that it can be used in any situation (eg, on a person’s own smartphone) and allows for the documentation of changes in momentary internal states, such as stress, tension, or mood [23]. Time-use research using EMA has primarily examined individual activities (eg, physical activity [24,25]), yet this methodology has rarely been used to study time-use patterns of different daily activities [26,27].

This is a key research gap because time-use survey techniques differ in that standard survey questionnaires more accurately and reliably measure longer-lasting activities such as paid work, whereas EMA through smartphones is more reliable for shorter-term and less frequent activities (eg, errands and housework) [27]. This means that research questions concerning gender differences in time-use patterns (especially with regard to unpaid activities such as housework and childcare) can be measured more precisely with EMA. Nevertheless, this has rarely been implemented in previous research.

Moreover, introducing EMA to daily activities might focus one’s attention on the present moment, thus increasing state mindfulness. Being present and bringing attention to the present moment is a key component of mindfulness [28,29]. Mindfulness is positively associated with higher levels of life satisfaction, optimism [30], and empathy [31]. It is also negatively associated with, for example, neuroticism [31,32] or difficulties in emotion regulation [33]. Hence, it is likely that regular reflection of and recording of the current activity and associated affect as a momentary and passing state depicts a form of mindfulness exercise [34]. As an exploratory hypothesis, the association between trait, state mindfulness, and daily activities will be examined.

### Research Questions

The planned study will address the relationship between time-use patterns, quality of life, and mindfulness, as well as momentary well-being and stress, with parents as the target group. The theoretical models and factors describing this relationship often neglect the type, duration, and sequence of activities; therefore, they will be complemented using the EMA method.

The EMA method has been used in the target group of parents only to measure specific activities (eg, activities with their own children like supervision and playing together) [35], whereas the measurement of global, daily time use combined with well-being has been used in this context only using student samples [26,27]. Thus, the feasibility of using the EMA methodology with a sample of parents will be examined.

Further, it will be investigated whether the monitoring of daily time use coupled with well-being and stress leads to a change in quality of life and mindfulness. In order to exclude the possibility that these daily mindfulness exercises alone can lead to a change in these constructs, a control group—that does not complete the daily surveys but only the baseline and follow-up questionnaires—will be included.

In summary, the following research questions are examined:

1. How do parents assess the feasibility of the study design?
2. To what extent do mothers’ and fathers’ time use influence well-being and feelings of stress, and vice versa? Does social support represent a mediator for the above association? Do gender, gender role beliefs, and work-family conflict represent moderators of the abovementioned associations?
3. To what extent does monitoring one’s own time use, well-being, and stress perception influence (1) the quality of life and (2) mindfulness?

## Methods

### Study Design

As this is a pilot study, a quasi-experimental, nonrandomized controlled trial with 3 data collection methods (ie, an online questionnaire [baseline and follow-up], an EMA survey, and interviews) was chosen. The intervention group will participate in the online questionnaires and EMA surveys, while the control group will only participate in the online questionnaires. A subsample of the intervention group will be invited to participate in interviews. The added benefit of recruiting a control group is to investigate whether daily monitoring of time use, well-being, and stress levels has an impact on overall quality of life and mindfulness.

### Participant Characteristics

#### Sample Description

The sample consists of fathers or mothers of at least 1 biological or adopted child who is younger than 18 years of age and living in the household. Participants are included irrespective of sexual orientation and relationship status. A completed informed consent form is also a requirement for inclusion in the study.

The following exclusion criteria will be applied: if participants (1) are younger than 18 years of age, (2) have one or more children with a chronic illness or disability, (3) have one or more foster child or children, and (4) do not own an Android smartphone (intervention group only). Due to technical reasons, only Android users can be included in the intervention group, and since families with a child with a chronic illness differ considerably in their daily routine from families without [36], we decided to exclude them.

#### Sample Size

The minimum sample size for the intervention group was calculated using G*Power (version 3.1.9.6; Erdfelder, Faul, and Buchner; ANOVA, repeated measures, within group, effect size [well-being]=0.25, α=.05, power=0.80, and correlation among repeated measures=0.5), and the target sample size is 64 participants.

For the control group, the target sample size is 17 participants. The sample size was also calculated using G*Power (ANOVA, repeated measures, within-between group, effect size [mindfulness]=0.25, α=.05, power=0.80, and correlation among repeated measures=0.5).

Regarding the number of participants for the interviews, the determination of the number of cases is based on the theoretical concept of data saturation [37]. This means that the sample size should be chosen according to how many interviews are necessary to provide significant new insights into the subject of the study [38,39]. Recommendations on the number of interviews to conduct until data saturation is reached vary from 5 to 60 interviews [37]. Because data saturation in homogeneous samples can be achieved with 6 to 17 cases [37,40], the target subsample size for interviews for this study is 6-17 participants.

### Incentives

#### Intervention Group

Participants will be offered an incentive of €35 (US $37.91) for participating in the questionnaires and daily EMA surveys, with an additional €15 (US $16.25) if they complete at least 80% of the daily surveys (ie, €50 [US $54.15] in total). If participants in the intervention group also take part in an interview, they will be offered an additional €10 (US $10.83).

#### Control Group

Participants taking part in the control group, that is, the baseline and final questionnaire, will be offered an incentive of €20 (US $21.66).

### Procedure and Measures

#### Participant Recruitment and Inclusion (T0)

**Figure 1:**
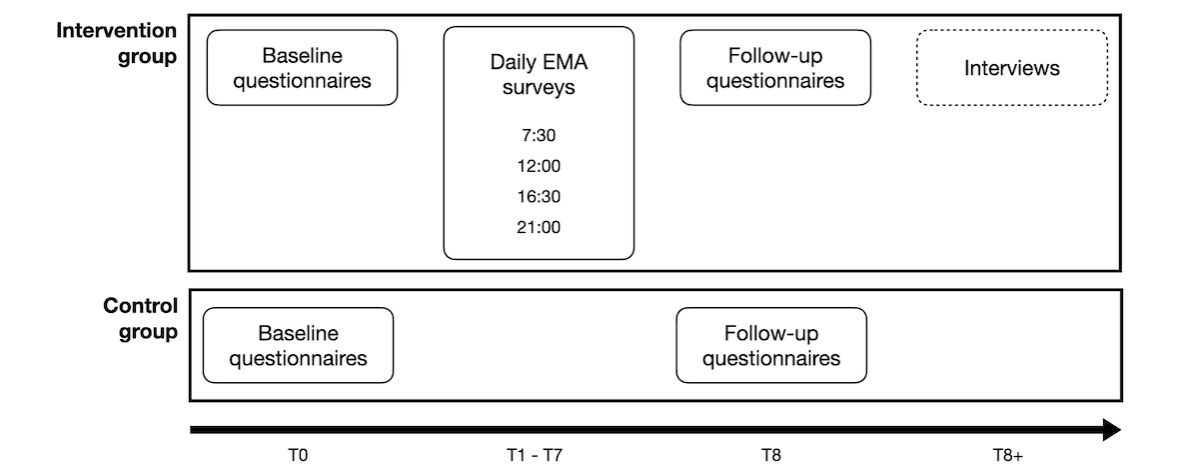
Overview of data collection. EMA: ecological momentary assessment.

Recruitment will take place through the snowballing method, in which the study will be advertised by means of leaflets through, for example, contacts with parents, parent groups, and relevant organizations (eg, family service centers and counseling centers). An overview of the data collection procedure can be found in Figure 1. An overview of the measures used in the questionnaires and during the EMA surveys can be found in Table 1. See the preregistration for the full questionnaires and interview guides [41].

The study will be advertised through leaflets. These contain a link (eg, by means of a QR code) to a short questionnaire, in which the contact information of the interested parties is collected. This questionnaire is carried out on an online survey platform (eg, SoSci Survey).

Afterward, an appointment is made by phone or email to introduce the study. This takes place digitally (eg, by telephone or video call). First, participants are informed about the study procedure and design. The inclusion and exclusion criteria are checked, and if excluded, they are informed of the reasons for their nonparticipation. If participants do not own an Android smartphone but fulfill all other inclusion criteria, they will be invited to the control group instead. If participants meet the inclusion criteria, they will be asked to download the movisensXS app (movisens GmbH; the survey tool of the EMA part of the study) on their personal smartphone and receive an introduction to its use. During the conversation, they will be given the opportunity to ask questions about the background and the study process. Participants are then sent an email with the link, where they provide written, informed consent. Only then can they access the links to begin the study.

**Table 1:** Overview of the measures for the questionnaires and ecological momentary assessment (EMA) surveys.

Survey time point or variable and questionnaires		T0	T1-T7	T1-T7 end-of-day questions	T8
**Questionnaires**					
	Expected feasibility^a^	✓			
	Sociodemographic background^a^	✓			
	Gender role beliefs^a^	✓			
	Work-family conflict^a^	✓			
	Social support^a^	✓			
	Quality of life, well-being, and stress^a^	✓			✓
	Trait mindfulness^a^	✓			✓
	Feasibility^b^				✓
**EMA surveys**					
	Current well-being and stress^b^		✓		
	Time use (current and since last survey)^b^		✓		
	Type of day^b^			✓	
	Daily social support^b^			✓	
	State mindfulness^b^			✓	

^a^Relate to both the intervention and control groups.

^b^Relate only to the intervention group.

#### Baseline Questionnaires (T0)

The baseline questionnaires will also be conducted on an online survey platform (eg, SoSciSurvey). Participants then complete the baseline questionnaires (T0); both the control and intervention groups will participate in the baseline questionnaire survey.

Participants will be asked, through open-ended questions, about the expected feasibility of the EMA surveys as well as their sociodemographic background (eg, age, gender, education and employment status, household income and size, partnership status, and number and age of children). In addition, gender role beliefs are surveyed using the Scale of Gender Role Attitudes [42], and work-family conflict is surveyed using the Work and Family Conflict Scale [43]. Further, social support is measured with the brief form of the Perceived Social Support Questionnaire (F-SozU K-6) [44], quality of life is measured with the World Health Organization-Quality of Life-8 (WHO-QoL-8) [45], affective well-being is measured with 3 items to assess positive affect (happy, relaxed, and satisfied) and 2 items to assess negative affect (nervous, downhearted, and worried) [46], and stress is measured with the global measure of perceived stress [47]. Finally, trait mindfulness is assessed with the Five Facet Mindfulness Questionnaires (FFMQ-D) [48].

#### EMA (T1-T7)

##### Overview

The intervention group will begin the EMA surveys after the baseline questionnaire. The EMA part of the study will be conducted through the movisensXS platform, which specializes in ambulatory assessment software. For graphic examples of the user interface, refer to movisensXS [49].

Over a 7-day period, participants will be surveyed 4 times per day (T1-T7). The survey times are 7:30 AM, 12 PM, 4:30 PM, and 9 PM, plus a tolerance period of 30 minutes; any later and the survey time point is considered not completed. Participants will receive push notifications at each survey time point and will be asked to complete the appropriate measurement instruments. The daytime surveys take approximately 5 minutes to complete, while the evening survey takes approximately 8 minutes.

Since 1 of the main objectives of the pilot study is to test the feasibility of the EMA design in a parent sample, an initial evaluation of feasibility (research question 1) will be conducted with the data from the first participants (n≤16). For this, the questions addressing feasibility, that is, the open-ended questions in the follow-up questionnaires and the interviews, will be examined. Subsequently, the study design (eg, survey times, item order, and item wording) will be adjusted if necessary. Therefore, a larger number of participants will be recruited for piloting.

##### EMA Questions

In the EMA surveys, participants are asked about their current well-being, feelings of stress, and current activities, as well as activities since the last survey at each of the 4 daily survey time points.

Well-being and stress perception are asked comparably to the baseline questionnaire, only with reference to the current state. Affective well-being is assessed with 4 items [46], cognitive well-being is assessed with the life satisfaction item of the WHO-QoL-8 [45], and stress perception is assessed with the global measure of perceived stress [47].

Participants can select the following categories, according to Tomczyk et al [6], to determine activities: (1) work; (2) housework, errands, and repairs; (3) childcare and organizing; (4) unpaid care work; (5) study and education; (6) leisure, physical activity, and hobbies; and (7) self-care. Multiple selections of activities at 1 point are possible. Participants first report the current activity as well as when they have been doing this activity. Then they are asked to recreate the sequence since the last survey point, that is, “we are interested in what you did between the last survey and now,” “what did you do afterwards,” and “for how long?”

In the last survey of the day, in addition to the questions mentioned above, so-called “end of day” questions are asked. The type of day (eg, ordinary working day or vacation day) is asked using the item from the Harmonised European Time Use Surveys [50]. The received and desired social support of significant others [51] is surveyed. Finally, the 3 dimensions of state mindfulness—present-moment attention, acting with awareness, and nonjudgmental acceptance—are measured using the Multidimensional State Mindfulness Questionnaire [52].

#### Follow-Up Questionnaire (T8)

One week later, both the intervention group and the control group will receive an email with the link to the follow-up questionnaire. This will take place on the same online survey platform (eg, SoSciSurvey).

At follow-up, social support (F-SozU K-6) [44], quality of life (WHO-QoL-8) [45], affective well-being [46], stress [47], and trait mindfulness (FFMQ-D) [48] will be measured again. The intervention group will be further asked questions about the feasibility of the EMA survey. For this, previous studies were used as a reference (eg, [26,53,54]). Example items include “Did using the EMA survey make them more aware of their thoughts, feelings, and behaviors?” (inspired by Businelle et al [53]) or “In my opinion, the questions were understandable” (inspired by Spook et al [55]).

#### Interviews (T8+)

Finally, a subsample of the intervention group will be invited for an interview (eg, by telephone or video call) lasting 20-30 minutes. The interview guide was developed using previous qualitative research focusing on usability and feasibility evaluations [56,57] and adapted to the present procedure (eg, feasibility of specific time points).

The interviews will address the feasibility of the EMA survey method and study design in more detail. The structure and content of the interviews are as follows:

1. General assessment of the study design
2. Time expenditure and integration into everyday life
3. Contents of the EMA surveys
4. Effects of the study
5. General and conclusion

### Analyses

Various between-person factors (eg, age, gender, family composition, and work situation) act as descriptive measures of the sample.

The quantitative material of the questionnaire survey will be analyzed using descriptive analyses to answer the first research question (feasibility of the design). On the other hand, for the evaluation of the open-ended questions in the baseline survey and the interview material, the content analytic approach of Kuckartz [58] is applied. The analysis and coding of the texts or transcripts will be done independently by 2 people. The text sequences or sense units are coded, and categories are derived from these contents or assigned to existing categories. The resulting category systems enable structured processing of the contents.

To answer the second research question (relationship between time use and well-being and stress), variance-analytic and regressive models with repeated measures and multilevel design will be used to analyze within-person change in baseline and follow-up questionnaires as well as to model the sequence of time use, well-being, and stress within the 7-day measurement. In addition, moderator and mediator variables will be considered (eg, gender role beliefs, social support, and work-family conflict).

To answer the third question (influence of monitoring of own time use, well-being, and stress perception on quality of life and mindfulness), within- and between-person models will be used to look at potential differences in the intervention and control groups.

### Ethical Considerations

The pilot study was approved by the Ethics Committee of the University Medicine Greifswald in July 2022 (BB 113/23) and will be conducted in accordance with the ethical standards laid down in the 1964 Declaration of Helsinki, its later amendments, or comparable ethical standards. This study has been preregistered on the Open Science Framework in November 2023 [59]. Informed, verbal as well as written consent is obtained from each participant; they are informed about how their data will be used and stored, as well as about their pseudonymized participation, and their ability to withdraw from the project at any time without any repercussions will be made clear to them. Ethical guidelines and General Data Protection Regulation will be followed concerning data usage and storage.

### Expected Outcomes

We anticipate that participants will regard the daily EMA as feasible. Particular daily time-use patterns (eg, high paid and unpaid workload) are expected to be related to lower well-being, higher stress, and health-related quality of life. These associations are expected to be moderated and mediated by factors such as gender, gender role beliefs, work-family conflict, and social support. Participants in the intervention group are expected to show higher values of mindfulness, well-being, and health-related quality of life and lower stress. Furthermore, we will report on dropout rates and reasons for dropout.

## Results

Patient recruitment started in November 2023 and ended in mid April 2024. Data analysis commenced in mid April 2024.

## Discussion

This study aims to provide valuable insights into the feasibility of using the EMA method with a sample of parents. A strength of the study design is the mixed methods approach, which combines online questionnaires, EMA surveys, and qualitative interviews. In the online surveys and qualitative interviews, we survey parents’ acceptance, challenges, barriers, facilitators, and feasibility of implementing EMAs in their everyday routine. This provides quantifiable feedback regarding the feasibility of the study design and is enriched by a subgroup of participants’ being able to speak in greater detail and with their own words. Studies with student samples suggest that daily diary methods more accurately and reliably measure longer-lasting activities such as paid work, whereas EMA through smartphones is more reliable for shorter-term and less frequent activities (eg, errands and housework) [27]. We examine how feasible the EMA method is in a sample (ie, parents) that is highly stressed, faces a large burden, and is notably strapped for time. Based on our findings, we will be able to make recommendations for the effective use of EMAs in parent samples.

The study is designed to provide insights into the associations between time use, well-being, and stress, as well as potential moderating and mediating factors such as gender role beliefs and work-family conflict. By using sequence analysis, we can determine which activities have a significant impact on stress and well-being. This information can then be used to recommend daily practices from a prevention perspective. Additionally, we recognize the relevance of gender issues and aim to explore gender and gender role-based differences in daily activities and how these relate to cognitive and affective states. Furthermore, we explore the effect of activity tracking on mindfulness and whether daily tracking can assist in guiding attention and promoting mindfulness or, conversely, increase perceived stress, particularly in individuals who are already stressed.

However, it is important to acknowledge the limitations of this study. It is a pilot study, and therefore, any associations found between variables should be interpreted with caution. Future research could explore dyadic effects between partners and also examine partner relationships. A further focus could be on specific target groups, such as individuals with high stress levels (eg, parents of children with a disability or chronic illness), who may particularly benefit from time management training interventions or planning interventions.

Overall, this study aims to contribute valuable insights into the feasibility of using EMAs and the potential benefits of activity tracking in various aspects of daily life. The implications of this study may contribute to the design of future mobile apps that track and use daily activities for purposes such as improving well-being, activity planning, or time management.

## Abbreviations

EMA: ecological momentary assessment
FFMQ-D: Five Facet Mindfulness Questionnaires
F-SozU K-6: Perceived Social Support Questionnaire
WHO-QoL-8: World Health Organization-Quality of Life-8
